# A target of potential RELAvance in inflammatory breast cancer

**DOI:** 10.18632/oncotarget.17109

**Published:** 2017-04-14

**Authors:** Bedrich L. Eckhardt, Naoto T. Ueno

**Affiliations:** Department of Breast Medical Oncology, Section of Translational Breast Cancer Research, and The Morgan Welch Inflammatory Breast Cancer Research Program and Clinic, The University of Texas MD Anderson Cancer Center, Houston, TX, USA

**Keywords:** inflammatory breast cancer, NF-κB, DSF-Cu, tumor emboli, high-throughput screening

Inflammatory breast cancer (IBC) is an insidious form of aggressive breast cancer that accounts for approximately 8-10% of all breast cancer-related deaths in the United States [1, 2]. Commonly IBC does not present as a lump, but instead as a severely localized carcinoma that has a high local recurrence rate and is extremely metastatic [3]. Patient survival has improved through the use of a multimodality strategy consisting of neoadjuvant chemotherapy (or targeted drugs if the tumor is positive for HER2 or estrogen receptor), radical surgery, and adjuvant radiation. However, IBC frequently becomes resistant to therapy, and ultimately patients die of progressive disease. Thus, there is an urgent need to better understand the molecular pathology of IBC so that better therapies can be devised.

One pathological hallmark of IBC is the occurrence of tumor emboli (tumor aggregates) in the dermal lymphatics, but they are not required as a diagnostic criterion. These emboli obstruct homeostatic drainage mechanisms and have been implicated in the elevation of localized inflammation and tumor spread [1]. Indeed, Arora *et al.* [4] discovered that RELA protein (also called, nuclear factor NF-kappa-B p65 subunit) - a major subunit of the NF-κB complex - was expressed in the tumor emboli of all IBC patient specimens. The detection of RELA in tumor emboli suggests heightened NF-κB signaling, a pathway associated with cancer cell survival, proliferation, and response to inflammation [5]. As could be expected, a downstream target of RELA, XIAP (X-linked inhibitor of apoptosis protein), is a potent anti-apoptotic protein that was similarly overexpressed in a majority of tumor emboli. Taking these results together, it was proposed that emboli formed in IBC could induce inflammation and trigger NF-κB activation in cancer cells, leading to their enhanced survival.

While inflammation may be a major determinant for IBC etiology and progression, therapeutic strategies designed to target inflammation-response pathways are yet to be evaluated. Toward answering this, the present study by Arora *et al.* investigated the potential of various drug compounds to affect the pathogenesis of IBC. A multiparametric, high-content analytical method was developed to evaluate the viability and therapeutic response of IBC emboli formed *in vitro*. Fluorescent labeling of three-dimensional IBC cultures enabled digital quantification of spheroid growth, morphology, and survival characteristics. Using these metrics, various NF-κB inhibitors were tested for their ability to perturb emboli formation.

Inactivation of NF-κB with broad-spectrum inhibitors blocked several aspects of emboli biology, including altered spheroid architecture and decreased viability. The lead compound, disulfiram, an FDA-approved compound for alcoholism, drastically prevented IBC tumor emboli formation *in vitro* when it was chelated to copper (DSF-Cu). Supporting their finding, other studies have demonstrated the effectiveness of DSF-Cu to induce apoptosis in IBC cancer cells (while sparing healthy tissue), leading to impaired tumor growth in animal models [6]. The cytotoxic properties of DSF-Cu pertain not only to inhibition of NF-κB, but also to its ability to block aldehyde dehydrogenase 1 (ALDH1) activity - an enzyme linked to cancer stem-like cells, which are aberrantly elevated in IBC tumors [7]. Similarly, DSF-Cu can functionally block DNA repair pathways and enhance the effects of DNA alkylating agents and radiation, thus making it an exciting compound that warrants clinical testing as an adjuvant therapy. Toward this, a Phase I/II clinical trial (NCT02715609) is currently investigating the maximum tolerated dose of DSF-Cu in patients with glioblastoma. Before use in IBC, adjuvant DSF-Cu therapy will require several clinical studies that would aim to test: dose scheduling, effectiveness in combination with standard of care neoadjuvant therapy, selection of appropriate patient populations and evaluation of rationalized molecular markers of therapeutic response.

A greater understanding of the molecular aspects and selection of efficacious therapies that destroy tumor emboli could provide new therapeutic strategies for IBC. Excitingly, this multiparametric spheroid/emboli assay could be extended to screen large drug libraries for other compounds that actively destroy IBC emboli. This platform could also be modified to longitudinally monitor emboli formation and response to therapy over time through the use of stably integrated fluorescent proteins. Further, it could be adapted to perform high-throughput, functional genomic screens that assess the genetic determinants of emboli formation and survival through systematic gain/loss of gene expression studies. Correspondingly, the question of how tumor emboli overcome drug resistance could be answered through synthetic lethality screens that pair drug and functional genomic approaches. Collectively, the platform developed by Arora *et al.* holds great promise for the pursuit of new drug therapies for IBC, as evidenced by the relatively high expression of NF-κB in IBC emboli and the successful evaluation of the RELA inhibitor DSF-Cu.

## References

[1] Fouad TM (2014). Adv Exp Med Biol.

[2] Masuda H (2013). Ann Oncol.

[3] Fouad TM (2015). Breast Cancer Res Treat.

[4] Arora AJ (2017). Oncotarget.

[5] DiDonato JA (2012). Immunol Rev.

[6] Allensworth JL (2015). Mol Oncol.

[7] Piras F (2011). Eur J Histochem.

